# Patient question set proliferation: scope and informatics challenges of patient question set management in a large multispecialty practice with case examples pertaining to tobacco use, menopause, and Urology and Orthopedics specialties

**DOI:** 10.1186/s12911-016-0279-2

**Published:** 2016-04-12

**Authors:** Sarah J. Vande Loo, Frederick North

**Affiliations:** Mayo Clinic, 200 First Street SW, Rochester, MN 55905 USA; Primary Care Internal Medicine, Mayo Clinic, 200 First Street SW, Rochester, MN 55905 USA

**Keywords:** Informatics, Patient-generated health information, Interoperability, Patient phenotype, Patient questionnaires, Patient question sets

## Abstract

**Background:**

Health care institutions have patient question sets that can expand over time. For a multispecialty group, each specialty might have multiple question sets. As a result, question set governance can be challenging. Knowledge of the counts, variability and repetition of questions in a multispecialty practice can help institutions understand the challenges of question set proliferation.

**Methods:**

We analyzed patient-facing question sets that were subject to institutional governance and those that were not. We examined question variability and number of repetitious questions for a simulated episode of care. In addition to examining general patient question sets, we used specific examples of tobacco questions, questions from two specialty areas, and questions to menopausal women.

**Results:**

In our analysis, there were approximately 269 institutionally governed patient question sets with a mean of 74 questions per set accounting for an estimated 20,000 governed questions. Sampling from selected specialties revealed that 50 % of patient question sets were not institutionally governed. We found over 650 tobacco-related questions in use, many with only slight variations. A simulated use case for a menopausal woman revealed potentially over 200 repeated questions.

**Conclusions:**

A group practice with multiple specialties can have a large volume of patient questions that are not centrally developed, stored or governed. This results in a lack of standardization and coordination. Patients may be given multiple repeated questions throughout the course of their care, and providers lack standardized question sets to help construct valid patient phenotypes. Even with the implementation of a single electronic health record, medical practices may still have a health information management gap in the ability to create, store and share patient-generated health information that is meaningful to both patients and physicians.

**Electronic supplementary material:**

The online version of this article (doi:10.1186/s12911-016-0279-2) contains supplementary material, which is available to authorized users.

## Background

The Office of the National Coordinator (ONC) for Health Information Technology defines patient-generated health information (PGHI) as a health-related data created, recorded, gathered, or inferred by or from patients or their designees to help address a health concern [1, 2]. Examples include health or treatment history, family history, symptoms, lifestyle information, preventive or chronic disease management, or biometric values taken through home monitoring devices [3]. This information augments clinical data found in the medical record to provide a more comprehensive picture of a patient's health [4]. Benefits of PGHI include increased patient engagement and satisfaction, reduced costs, and improvements in quality, care coordination, and patient safety [1, 5, 6]. The ONC Technical Expert Panel on patient-generated health information makes a point of stating that PGHI is evolving to encompass data from home monitoring equipment and other patient devices [1, 2]. This manuscript, however, is confined to information that is obtained from patient question sets.

Patient-generated health information can be obtained via question sets delivered to patients before or after appointments, during an episode of care, or on an ongoing basis to measure changes in health status. Within a large, multispecialty practice, there can be hundreds of such question sets in which the information is collected, stored and managed through various means. Without organizational oversight, standardization and shared data models, specialty areas and groups are unable to leverage data collected from multiple clinical areas, and moreover, have limited awareness of what is being asked when and by whom. Despite the implementation of a single electronic medical record (EMR), medical practices may still have a health information management gap in its ability to create, store and share patient-generated information that is meaningful to both patients and physicians [2, 4, 5].

Lack of regulation around the collection of PGHI causes repetitive requests for patient information, often with questions that vary only slightly. Insufficient coordination and governance of questions sets can reflect poorly on a health care organization and its data management. Also, the continual requests for information throughout the course of their care add to patient burden and may reduce response rates. Patients may erroneously believe that information provided in the context of an appointment are included in their medical record and therefore shared with other care providers, but this may not be the case. As question sets are proliferating, there is a concern over how many questions are repeated, and current literature is lacking to quantify the extent of the patient burden. Repetitious questions may not rise to the level of patient complaints but still could be an annoyance for patients and impact the collective perception of an organization.

Patient-generated health information is also important for construction of a patient phenotype. An informatics challenge has been to extract information from the EMR to generate a patient phenotype [7–11]. This phenotype information is obtained in part from patient-generated health information. For example, a tobacco exposure phenotype is constructed with patient-generated information about smoking exposure, types of tobacco use, and dates of onset and quit dates. Hripcsak and Albers point out how difficult this data is to collect, but questions around tobacco use could create structured data fields that would support a tobacco exposure phenotype that would be useful for clinical care as well as research [12]. Risk calculators for osteoporosis, lung cancer and heart disease all need patient-generated information about current and previous tobacco use. An EHR that automatically uses well-structured patient-generated tobacco information to calculate risk of lung cancer, heart disease and osteoporosis would significantly help clinicians at the point of care as well as in population management. In addition, a well-constructed patient phenotype will be an invaluable resource for investigators examining genotype-phenotype associations [13].

To better understand this health informatics challenge and the potential for data interoperability, we examined the following aspects of patient question set management to determine the scope of current state: estimated number of governed question sets in a shared forms database, variability and repetition of individual questions, and ungoverned question sets used by two specialty areas.

## Methods

### Study setting and design

This was an observational study done at Mayo Clinic, which is a large, integrated multispecialty practice with over 1.2 million outpatient visits and 130,000 hospital admissions per year. Mayo Clinic employs almost 60,000 physicians, nurses, scientists, and allied health staff at locations in the Midwest, Arizona and Florida, and it has over 150 divisions in multiple clinical specialties.

Our study design used a random sample of questionnaires accessible through a Mayo Clinic Forms and Publications database and a review of question sets from two specialty areas (Urology and Orthopedics). We also analyzed a simulated use case of a menopausal woman to estimate the number of repeated questions a patient could encounter through a single episode of care.

### Data collection

Data collection was done to estimate the number of governed and ungoverned question sets. Governed question sets are those that have official status at Mayo Clinic by being given a unique document ID and allowed universal dissemination through the clinic via the Forms and Publications database. Ungoverned question sets are not part of the Forms and Publications database and may be proprietary or in use within single clinical areas.

#### Capture of governed question sets

We identified governed questions by searching the Mayo Clinic Forms and Publications database, which includes 37,625 unique English language documents for active use across the Mayo Clinic sites. Within this group, there are 4626 documents when conducting full document searches on the terms *survey*, *assessment* and *questionnaire*; 210 are specifically tagged as patient questionnaires via the filter. Due to wide variance in how that filter is interpreted and applied to question sets in the database, we used a title search instead of the filter to determine the sample. Because we couldn't identify all of the patient question sets in the database, we queried the database using the term *questionnaire* in the title to find a sample. This convenience sample was further limited to a random sample of question sets that fit the query.

A total of 336 questionnaires were found in a search of the term *questionnaire* in the title. Of note, many patient question sets do not include the term *questionnaire* in their title, for example, Current Visit Information, Patient Family History, and Visual Analog Pain Scale, and therefore, are not included in the questionnaire title search and are not included in this sample.

**Fig. 1:**
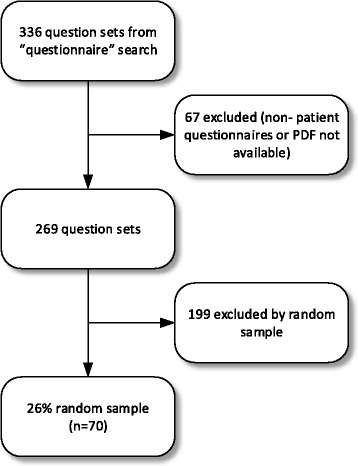
Question set flow from capture to random sample

From a complete review of all 336 question sets as obtained by the search criteria above, 67 were removed from the final dataset because they were not patient questionnaires or the PDF copy of the questionnaire was not available via the database search. After exclusion of these 67, the final question set count was 269. Using JMP Pro 11.0 for the randomization process, we created a random sample without replacement of 70 (26 %) from the set of 269 (Fig. 1).

#### Capture of ungoverned question sets

Ungoverned questions given to patients in two specialty areas (Urology and Orthopedics) were identified by interviewing staff and examining the processes to collect patient information in those two specialty areas. This was a convenience sample of two specialties that were selected based on ongoing institutional initiatives to identify ungoverned question sets. The specialties of Urology and Orthopedics were not identified *a priori* by the authors. The list of specialty questionnaires was compared against versions in the forms and publications database. Question sets delivered to patients that could not be matched up to any question sets in the database (that had the unique Mayo Clinic document IDs), were considered ungoverned from an institutional perspective. In our capture of total specialty areas question sets, we excluded question sets that are given to all patients such as Current Visit Information and Patient Family History question sets. When the specialty review was done, these types of all-purpose question sets were not included in the count as they could be answered as part of a separate appointment (and therefore not asked again at the specialty appointment). These questions sets are requested regardless of the appointment type (primary care, women's clinic, urology, orthopedics).

### Measures

In analyzing the governed sets to determine an estimated number, we counted questions in each of the questionnaires, excluding patient identification questions (patient name, patient ID, record number), patient notification information (address, email, phone number), and referring physician and notification of provider questions. Questions asking for general comments were also excluded. Apart from these exclusions, total question counts included all questions in the question sets that requested discrete patient information, regardless of whether a patient would answer every question. Also, if questions were repeated to collect similar data about separate events (for example, medical history, family history, pregnancies, medications), they were included in the total. From this total, we determined an average and were then able to estimate the number of officially governed patient questions.

#### Question variability: Tobacco-related questions example

To determine the variability of individual questions, an analysis was done to review the tobacco-related questions available in the forms and publications database and in the patient education database. Text searches were conducted to find tobacco-related questions within patient-facing question sets, for example *tobacco*, *smoking*, and *secondhand smoke*, were used to search question sets. Question text, answer text, answer types (e.g., multiple choice, free text, yes/no), question source, and identification numbers were noted in a spreadsheet. Question text and context was reviewed and the following categories were assigned to group the topics: behavior/treatment, usage, clinical, addiction, secondhand smoke, stressors/feelings, patient education and general. We assessed the question variability by counting the questions that were sufficiently different from others in product terminology (tobacco vs. spit tobacco vs. cigarettes), amounts of tobacco use (10 to 14 cigarettes per day vs. 10 to 19 cigarettes per day) and other differences in meanings. The question counts containing differences in meaning were placed into the eight categories described above.

#### Question repetition: Menopausal patient example

To determine the number of repeated questions that a single patient might encounter, we reviewed six questionnaires that could be given to a menopausal woman during a typical episode of care: current visit/patient family history, women's health questionnaire, menopausal health questionnaire, bone density and mammography questionnaires, and a research survey. We determined the topics and numbers for the baseline question set that is given to all patients, then reviewed how many of those questions were repeated in the subsequent requests for patient data.

### Analysis

We described the counts of question sets and individual questions with means, medians, ranges and standard deviations. We also used frequencies (percent) in describing categorical data for different types of tobacco-related questions.

## Results

### Estimated total governed questions

A random sample of 70 (26 %) patient question sets contained 5163 questions. The flow of this random sample is given in Fig. 1. The range of questions in the question sets was 4 to 485 with a mean of 74 and median of 39.5. Using this analysis, it is estimated that there are approximately 19,841 governed patient questions in the forms and publications database (see Table 1).

**Table 1:** Estimated number of governed questions

Count of eligible governed questionnaires	269
Sample size	70
Total questions in sample	5163
Range of question count per governed questionnaire	4–485
Mean no. of questions per questionnaire	73.76
Median no. of questions per questionnaire	39.5
Standard deviation	87.68
Estimated total number of governed questions	19,841

Information provided with the documents include item number, active/discontinued status, approved usage location (e.g., Rochester, Florida, Arizona), and availability to access. There is no indication of the intended use of the form, targeted patient group, or current usage by departments, site or physician group. The database also includes multiple versions of seemingly similar assessments, with no indication of which should be used when or for what purpose. For example, there are 10 question sets that assess levels of pain: Pain Assessment and Management, Pain Assessment Questionnaire, Pain Assessment Scale, Pain Scale Tool, Pain Survey, Visual Analog Pain Scale, Pain Clinic Daily Pain Diary, Pain Clinic Questionnaire, Pain Clinic Worksheet, and Pain Clinic Survey. While some of these question sets may have duplicative questions, others might serve a specific purpose for a provider or researcher and have deliberate differences. Without knowing more about how or when such question sets are used nor their purpose or targeted population, determinations around harmonizing such question sets cannot be made using the current tool set.

### Question variability

There is large variability in how questions are asked to collect patient data. In the group of tobacco-related questions that was analyzed, it was determined that at least 650 unique questions exist. The majority of these were about behavior/treatment (389 unique questions) and tobacco usage (129 unique questions) (see Table 2).

**Table 2:** Number of tobacco-related questions by category

Category of tobacco questions	Unique question count
Behavior/Treatment	389 (59.8 %)
Usage	129 (19.8 %)
Clinical	95 (14.6 %)
Addiction	14 (2.2 %)
Secondhand smoke	7 (1.1 %)
General/Other	7 (1.1 %)
Stressors/Feelings	5 (0.8 %)
Patient education	4 (0.6 %)
Total tobacco questions	650

Within the usage category, wide variations existed in the wording of questions, timeframes presented (e.g., tobacco usage in the last week, 30 days, 90 days or last year), question types (free text, yes/no, multiple choice), and answer groupings (e.g., 10 to 14 cigarettes, 10 to 19 cigarettes, 10 to 20 cigarettes). There were also variations seen in terminology (e.g., smokeless tobacco, spit tobacco, chewing tobacco, snuff) and frequent interchanging of the terms *smoking, cigarette smoking* and *tobacco use*. Examples are as follows: Do you currently smoke cigarettes or have you used other tobacco products in the past year? In your lifetime, have you smoked more than 100 cigarettes? Do you currently smoke cigarettes? In the past 7 days, have you smoked cigarettes? In the past 90 days, have you used any type of tobacco (cigarettes, cigars, pipe, snuff or chewing tobacco)? Has tobacco use been within the last 12 months? Do you now or have you ever used tobacco on a regular basis?

### Question repetition

From a patient's perspective, requests for information occur frequently, often with the same question asked multiple times during an episode of care. In our analysis of six patient question sets targeted toward menopausal women (see Additional file 1), we found that our Current Visit Information and Patient Family History baseline questionnaire included 294 questions within ten main topic areas (e.g., medication, allergies, review of systems/symptoms, past medical history, social history, lifestyle). Across the five subsequent questionnaires, a total of 218 questions or data points were repeated from that baseline. Frequently, repeated questions included history of pregnancies and live births, menstrual periods and menopausal questions, medical history (e.g., cancer, heart disease, osteoporosis), surgical history (e.g., hysterectomy, mastectomy), relationship status, and tobacco and alcohol use.

### Estimation of ungoverned question sets

Clinical areas may develop ungoverned question sets for use within their clinical areas, and these are not included in the forms and publications database. In an initial analysis of the Urology Department in Rochester, Minn., eight main questionnaire forms were used. Of these, 4 (50 %) were ungoverned, comprising of 248 total questions, ranging from 16 to 89 and an average of 62 per ungoverned questionnaire. In a sample reviewed from the Orthopedics area, there were 16 patient forms and 10 (63 %) were ungoverned with a total of 198 questions, ranging from 1 to 50 questions and an average of 20 per ungoverned questionnaire (see Table 3). Identifying these as ungoverned forms does not mean the question sets are not validated or evidenced-based, but rather that they are used exclusively by a single work unit or department and therefore have no broader access or visibility within the organization.

**Table 3:** Ungoverned questions in Urology and Orthopedics specialty areas

	Specialty area	
	Urology	Orthopedics
Total questionnaires	8	16
Count of ungoverned questionnaires	4	10
Count of ungoverned questions	248	198
Range of question count per ungoverned questionnaire	16–89	1–50
Mean questions per ungoverned questionnaire	62	20

## Discussion

There is an enormous amount of variability in the collection and storage of PGHI [1, 2, 5, 14]. Within a single organization there can be hundreds of question sets and thousands of questions that lack standardization and governance across clinical groups and care teams. There is little visibility into what PGHI is being collected when and by whom [14]. Typically, the data is not stored in a way that can be easily reused or shared, even within an organization that has an EMR [2, 14]. Patients may be asked the same question multiple times or pertinent questions may be missed.

The challenges are numerous when considering the flow of information between the patient and provider and the technology infrastructure that must be in place to support transmitting, receiving, documenting, storing and analyzing such data [1]. The 2014 JASON Data for Individual Health report [15] highlights the benefits and challenges of developing a health information technology ecosystem that standardizes patient-reported information collected beyond traditional means. This Learning Health System initiative is designed to improve individuals' health by linking traditional health care to new sources of relevant data, such as via digital or mobile data collection tools [15].

**Fig. 2:**
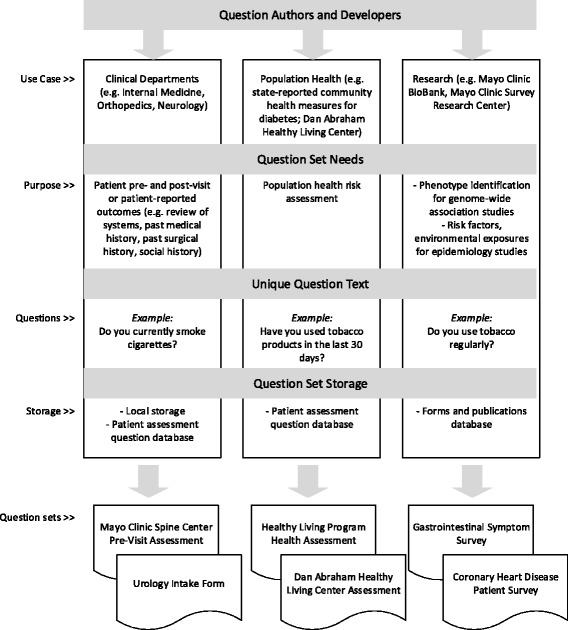
Contributing factors associated with the proliferation and variability of patient question sets

Through our research, the authors observed four major contributing factors associated with the proliferation and variability of patient question sets: question set needs, distinct question authors and developers, unique question text, and question storage (Fig. 2). First, the need for PGHI varies across physician groups, departments and specialty areas. Patient data may be necessary for population health risk assessment, research, patient reported outcomes, chronic disease management, and other purposes [1, 2, 14]. Multiple groups identify such needs and develop patient question sets to collect their data.

Second, large organizations may have disparate groups of authors and question set developers who conceptualize and produce the question sets and build discrete methods for delivering them. Authors may leverage questions from national sources [16–18] or well-known validated tools [19–21] or may develop unique or proprietary question sets. This leads to lack of question standardization and duplication of content.

Third, question text itself offers variability, especially when factoring in the number of authors and stated needs. A single question could have many slight variants even when the intent is to collect the same information, for example, current tobacco use status. Variations of terminology and interpretation of the data needs (e.g., "regular" tobacco use vs. "any" tobacco use vs. "current" tobacco use) leads to differences not only in the question text but, more importantly, in the data collected.

Last, separate storage locations inhibit shared access. Without greater awareness of what is already developed, authors create a new variation, or identical questions may be developed and stored in multiple locations because it is unknown that the question or question set exists elsewhere.

In the end, the burden of such variability and lack of overall coordination negatively impacts patient engagement and satisfaction [2]. Patients are asked to respond to repeated requests for data and to answer identical or similar questions throughout his or her continuum of care. If patients ignore such requests, then critical information is not collected, which can impact the quality and safety of patient care [5].

### Addressing the challenge

**Fig. 3:**
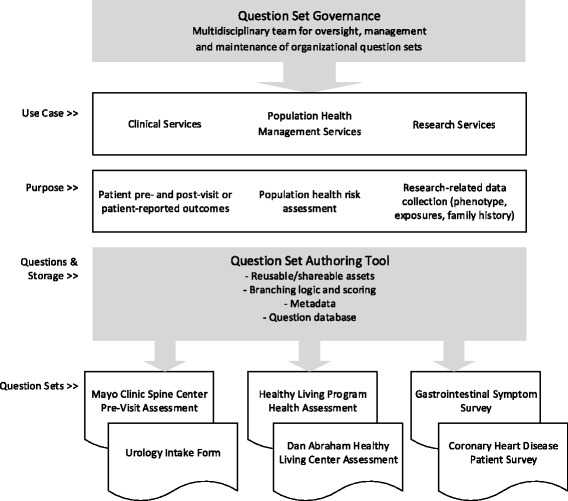
Model of question set management to enhance standardization and reduce variability of questions

Even with standardized questions and shared data solutions, question set development and management issues will likely persist. Within large organizations, there will likely be multiple question developers who have unique information needs that are stored in various databases. The key to governance within such challenges will be three-fold: 1) ability to author and store question sets to support central storage and mapping of data, 2) codification of the data to support interoperability, and 3) a centralized governance structure to facilitate shared development and maintenance of patient question sets (Fig. 3).

#### Question set authoring

Most of the question sets found in this study are distributed to patients via paper forms. Data collected is either not stored for long-term use or is stored in a local database or file system with limited access and shared use. A solution will require patient questions in a shared database accessible to all specialties in the practice. A solution will also need to include the logic and algorithms necessary to deliver the right question to the right patient at the right time. A central authoring tool and database will allow for the development, storage and delivery of reusable and shareable assets necessary to collect patient information [22]. A shared tool will also provide a broader awareness of what questions are already developed by whom and for what purpose. Metadata, attributes and other properties can be assigned to support question categorization and allow search of an institutional catalog of questions.

#### Interoperability

The digitization of question sets should support the central storage and mapping of patient data. Patient data collected through multiple delivery channels can be delivered to a central storage database and integrated into the EMR for improved clinical experiences [14, 22]. The structuring and codification of the patient data will ensure standardization and interoperability across platforms and facilitate a broader awareness of what patient information is available and for what purpose [5, 14, 23, 24]. As evidence of this, proposed rules in the 2015 HIT Certification Criteria for Meaningful Use Stage 3 assigns a larger role to the standardization of the collection of patient-generated health information. The proposal has identified a set of social and behavioral domains and standardized questions and answers with associated LOINC codes, for example, alcohol use (AUDIT-C) and PHQ2 depression screening, so that patient information can be collected in a consistent, standard way [24].

The codification of the patient data, for example, using LOINC or SNOMED CT industry codes [24–27], will allow for some textual variations between the question sets. This means, however, that such industry codes will need to provide enough granularity to accommodate the number of different ways that people want to collect and use the data, but enough generality so that patients aren't asked multiple times for the same information to account for minor textual differences. To date at our institution, industry codes are not applied to PGHI collected through patient question sets. However, to develop a patient phenotype that can be linked to genotypes both within the institution and across health care systems, standardized codes for this PGHI will need to occur [13, 28]. The PhenX Toolkit initiative led by RTI International and funded by the National Human Genome Research Institute is addressing some of this standardization [29, 30]. The PhenX Toolkit currently contains 474 measures in 23 domains that standardize the way data is collected. The PhenX Toolkit includes recommended, broadly validated measures, along with associated protocols for collecting the data. The measures and protocols have been selected and vetted by working groups of domain experts.

#### Centralized governance

Data collection tools should be centrally governed through a multidisciplinary team to ensure consistency across clinical groups, departments and units. A centralized oversight structure can facilitate the collaboration, development and maintenance of patient data collection tools and can advocate for the best patient experience [14]. The Precision Medicine Initiative Cohort Program, initiated by the National Institutes of Health, outlines the importance of standardization of data collection and baseline measurements required for the "precise measurement of molecular, environmental, and behavioral factors that contribute to health and disease" [28]. Furthermore, using EMR quality assessment information, data governance teams can also ensure that data is structured, codified and stored in a manner suitable for reuse for clinical research and phenotyping [12, 31]. Not only will patients be presented fewer repetitive questions, but clinicians and researchers will benefit from structured data that can generate more standardized patient phenotypes needed for genome association studies, risk calculators and other assessments [14].

### Limitations

This study has several limitations. We did not exhaustively examine all 269 of the convenience sample of patient question sets in the database and instead based our conclusions on a random sample of 70 from the larger set of 269. The convenience sample also does not represent the complete set of patient questionnaires in the database because many did not match our query term *questionnaire*. We also based the estimate of ungoverned patient question sets on our experience with only two specialties. Our methodology did not allow us to directly count repeated questions asked of patients during their clinic encounters. To do that would require direct observation of patients as they went through their visit itineraries that frequently include multiple specialty visits. Instead we wanted to give an example of the potential for repeated questions so we chose a common patient, a menopausal woman. Other patients could certainly have the potential for fewer or even more repeated questions. Finally, our study was conducted within a large, multispecialty practice and the scope of the issue may not generalize to other organizations, especially smaller clinics. We are continuing to learn about the barriers and benefits of large-scale governance of patient question sets. Our study is to demonstrate the scope of this challenge and how one institution is addressing the challenge rather than to advocate a specific governance model.

## Conclusion

Despite efforts to migrate to electronic medical records, gaps remain in the development and management of patient question sets. Although this analysis highlights several challenges in the collection, storage and sharing of PGHI at a large, multispecialty health care organization, the issues at hand are applicable to smaller, more focused health care facilities. This study shows major interoperability challenges of PGHI within a single EMR. As we look forward to increased interoperability, we will need to systematize PGHI collection across EMRs as well.

Our study of PGHI in a large multispecialty group practice shows some major challenges, but quantifying the full scope of the challenge is difficult due to lack of centralized content databases and indeterminate number of ungoverned question sets. The size of a health care institution, number of patients, number of specialty areas, and number of patient question sets add to the complexity and magnitude surrounding patient question set management.

The research gathered through this study serves as a gauge that suggests a large-scale problem. By moving toward an integrated system, with shared question sets and data models and common governance, we can learn more about optimal ways to request, store and share patient-generated health information that's meaningful to both patients and physicians.

## Ethics

No patient data was used in this study. Our question set review involved question sets and questions that were in use but not populated with any patient data. Because this research did not involve humans, human data or animals, ethical approval was not required. The Mayo Clinic Institutional Review Board (IRB) acknowledged that this study did not require IRB review.

## Consent

No consent was needed for this study.

## Availability of supporting data

The Mayo Clinic forms data set was used for this study but is not available to share.

## Additional file

Additional file 1: Potential repeated questions for a menopausal woman. Data gathered through an analysis of six patient question sets targeted toward menopausal women that shows the potential of repetitious questions. (PDF 27 kb)
