# Supplementary material for: Patient question set proliferation: scope and informatics challenges of patient question set management in a large multispecialty practice with case examples pertaining to tobacco use, menopause, and Urology and Orthopedics specialties
Source: BMC Med Inform Decis Mak. 2016 Apr 12;16:41. doi: 10.1186/s12911-016-0279-2 (PMC4828833; doi:10.1186/s12911-016-0279-2)
Supplement: Additional file 1: — Potential repeated questions for a menopausal woman. Data gathered through an analysis of six patient question sets targeted toward menopausal women that shows the potential of repetitious questions. (PDF 27 kb) [file 12911_2016_279_MOESM1_ESM.pdf]

Additional File 1. Potential repeated questions for a menopausal woman

**BASELINE:**<br>**Current Visit Information/Patient Family History**<br>(new patients, yearly thereafter)		**Women's Health Questionnaire (Internal Medicine)**		**Menopausal Health Questionnaire (Women's Health Clinic)**		**Bone Density Questionnaire (Radiology)**		**Mammography Questionnaire (Breast Radiology)**		**Community Survey of Women (Research Survey)**		**Total shared questions**
Sections	No. of questions	Examples of shared questions	No. shared questions	Examples of shared questions	No. shared questions	Examples of shared questions	No. shared questions	Examples of shared questions	No. shared questions	Examples of shared questions	No. shared questions	
Patient information and demographics	8	Patient ID, DOB, gender	3	DOB, gender	2	Patient ID, DOB, gender	3	Patient ID, DOB, gender	3	DOB	1	12
Medications	9	Oral contraceptives	1	Medications, doses, frequency	3	Osteoporosis medications, oral steroids	2	Oral contraceptives	1	Oral contraceptives	1	8
Allergies	12	N/A	0	Medications, reactions, other allergies and reactions	4	N/A	0	N/A	0	N/A	0	4
Review of systems and symptom assessment	77	Headaches, pain, sexual performance, sleep problems	4	Headaches, sexual performance, sleep problems, urination problems, joint pain	13	N/A	0	Nipple discharge, breast lumps	2	Headaches, sexual performance, sleep problems, joint or muscle pain, rapid heartbeats, anxiousness/nervousness	13	32
Past medical history	80	Pregnancy, live births, menstrual periods, cancer history, other conditions	17	Pregnancy, live births, menstrual periods, cancer history, other conditions	41	Rheumatoid arthritis, diabetes	2	Pregnancy, live births, menopause, breast cancer	4	Menstrual periods, menopause	2	66
Past surgical history	41	Hysterectomy, tubal ligation, cancer surgeries/therapies,	9	All surgical history (free text option)	41	Hip surgery, spine surgery	2	Mastectomy, breast biopsy, breast implants	3	Hysterectomy	1	56

Family history	44	Heart disease, stroke, cancer, osteoporosis	7	Heart disease, stroke, cancer, osteoporosis, depression, alcoholism, drug abuse	12	N/A	0	Breast cancer	1	N/A	0	20
Social history	7	Relationship status	1	Relationship status, employment, occupation	3	N/A	0	N/A	0	Relationship status, education status	2	6
Preventive, screening and immunizations	10	Mammogram, pap test, HPV	3	Mammogram, pap test, abnormal pap test	3	N/A	0	Mammogram	1	N/A	0	7
Lifestyle	6	Alcohol use	1	Tobacco and alcohol use	4	Tobacco and alcohol use	2	N/A	0	N/A	0	7
	294		46		126		11		15		20	218
